# COVID-19 Vaccine Uptake Through the Lived Experiences of Health Care Personnel: Policy and Legal Considerations

**DOI:** 10.1089/heq.2021.0027

**Published:** 2021-09-27

**Authors:** Rachel Gur-Arie, Zackary Berger, Dorit Rubinstein Reiss

**Affiliations:** ^1^ Berman Institute of Bioethics and Johns Hopkins University, Baltimore, Maryland, USA.; ^2^ Division of General Internal Medicine, Johns Hopkins University, Baltimore, Maryland, USA.; ^3^ The Esperanza Center, Catholic Charities, Baltimore, Maryland, USA.; ^4^ College of the Law, University of California, Hastings College of the Law, San Francisco, California, USA.

**Keywords:** health care personnel, COVID-19, vaccine uptake, equity, ethics

## Abstract

**Purpose:** To investigate whether coronavirus disease 2019 (COVID-19) vaccination campaigns targeted at health care personnel (HCP) in the United States have addressed the lived experiences of HCP on the frontlines of the COVID-19 pandemic and to analyze policy and legal considerations for improving COVID-19 vaccine uptake among HCP.

**Methods:** We conducted a literature and policy review to explore the lived experiences of different occupational groups of HCP on the frontlines of the COVID-19 pandemic—physicians, nurses, trainees, and nonclinical essential workers—in relation to ongoing COVID-19 vaccination campaigns. Finally, we discuss policy and legal considerations to improve the state of HCP COVID-19 vaccine uptake as the pandemic progresses.

**Results:** COVID-19 vaccination campaigns have not achieved consistent high uptake among HCP for many reasons, including vaccine hesitancy, personal, professional considerations, and equity-rooted challenges.

**Conclusion:** HCPs lived experiences during the COVID-19 pandemic reveal meaningful impediments to their COVID-19 vaccine uptake. We suggest that health care systems minimize inequity inherent in existing vaccination campaigns by providing financial and social support to HCP to raise HCP COVID-19 vaccine uptake.

## Introduction

The coronavirus disease 2019 (COVID-19) pandemic continues. Although multiple COVID-19 vaccines have been approved and global vaccination is ongoing, inequity and vulnerability continue to pose a disproportionate burden to certain communities and health care systems, despite vaccine rollout in many places.^1,2^ Many COVID-19 vaccination campaigns are experiencing logistical and communication challenges, impeding the speedy equitable distribution of available vaccines.^3^

Health care personnel (HCP), defined by the Centers for Disease Control and Prevention (CDC) in this context as “persons serving in health care settings who have the potential for direct or indirect exposure to patients or infectious material,”^4^ have been prioritized for COVID-19 vaccination. This prioritization is reflected in multiple vaccine allocation plans developed by public health professionals and bioethicists. According to the CDCs framework, prioritizing HCP for COVID-19 allocation is rooted in three characteristics of this group^1^: they themselves are at high risk for contracting COVID-19,^2^ they are doing essential work during the pandemic, and^3^ they are a source of COVID-19 transmission.^4^

As COVID-19 vaccination continues, discussion has broadened beyond prioritization to access and uptake. Distributing COVID-19 vaccine among HCP is proving to be a significant challenge, particularly in the United States.^5,6^ HCP continue to work long hours in under-resourced, overcrowded health care settings at the frontlines of the COVID-19 pandemic.^7^ While immense psychological, physical, and mental burdens are routine side effects of their profession, the emergency nature of the pandemic has stretched them beyond their limits.^8^ Multiple factors contribute to poor vaccine uptake among HCPs. Their occupational experience during COVID-19 is obviously significant. Additionally, documented vaccine hesitancy,^9,10^ decentralized health care, and fragmented policies in states and other jurisdictions also contribute to low vaccine uptake.

There are little data on the uptake of COVID-19 vaccines among HCP by occupational group in the United States. However, a study of COVID-19 vaccine uptake among more than 23,000 publicly employed HCP in the United Kingdom found uptake rates of more than 80% for all occupational groups.^11^ These occupational groups included administrative or executive staff, nurses or health care assistants, doctors, midwives, specialist staff, estates, porters, or security, pharmacists, health care scientists, and “other.”^11^ Given the United Kingdom's strong, centralized health care system (The National Health Service^12^), these uptake rates should not be extrapolated onto other contexts—particularly the United States' fragmented health care system.

The present analysis explores the legal and ethical consequences of these selected variables to shed light on potential directions for raising HCP COVID-19 vaccine uptake.

## COVID-19 Lived Experiences Among HCP

HCP of varying disciplines have been pushed beyond their physical and psychological limits during the COVID-19 pandemic, suffering from heightened exhaustion, insomnia, tremendous workloads, weak institutional support, daily ethical dilemmas, depression, anxiety, and, ultimately, burnout.^13,14^ Although these symptoms cross occupational lines among HCP, experiences of COVID-19 within a given population, such as HCP, are heterogeneous.^15^ Such nuances contribute to unique narratives, experiences, and vulnerabilities that should be recognized when implementing COVID-19 vaccine campaigns for HCP.

### Physicians

Physician leadership has guided the global fight against COVID-19 for over a year. Their lived experiences and challenges throughout COVID-19, characterized by shortages of personal protective equipment (PPE), workforce, and institutional policies of “do not resuscitate” COVID-19 patients, have presented unprecedented challenges that many have had to learn to manage in real time.^16^ Death resulting from occupational infection of COVID-19 is the ultimate price that many physicians have paid; insufficient PPE is frequently cited as a contributor.^17^ This contributes to mistrust among HCP in health care systems and institutions, which they work for.

Before the COVID-19 pandemic, almost half of all physicians were estimated to burnout, with higher burnout rates documented for women.^16,18^ The heroization of physicians during COVID-19 has contributed to a complicated public narrative in which physicians may feel hesitant to speak up against poor working conditions and response to the pandemic.^19^ But physician willingness to work in a pandemic is not without limits. A study conducted in response to the 2009 swine flu pandemic showed that only 25% of physicians believed that there was a duty to work when the work would pose risk to themselves or their family.^20,21^ While the prioritization of physicians to get vaccinated against COVID-19 is important, poorly implemented vaccination campaigns could quickly close the window of opportunity to regain the trust lost among physicians in health care systems throughout the COVID-19 pandemic.

### Nurses

Nurses spend the most one-on-one time with COVID-19 patients.^13,22,23^ As a result, nurses are often at increased occupational risk for COVID-19 infection in comparison to other groups of HCP.^22^ During the COVID-19 pandemic, nurses have taken on leadership roles, which are central to balancing staff and patient care, and, as a result, added professional burden.^24^ A qualitative study of nurses in China, conducted early on in the pandemic, suggested four themes that encompass nurses' psychological experience during a peak of COVID-19 infection and uncertainty^1^: a significant number of negative emotions,^2^ varied coping and self-care,^3^ growth under pressure, and^4^ positive and negative emotions occurring simultaneously or progressively.^23^ Similar themes have been expressed within an American context.^24–27^

Nurses navigate a spectrum of shifting priorities, personal and professional boundaries, and well-being.^27^ During the COVID-19 pandemic, it is not uncommon for nurses to cry or experience attacks on shift, while feeling institutional pressure to stay quiet to stay employed.^24^ An additional defining source of anxiety for nurses during the COVID-19 pandemic, unique compared with other professions, is the fear that all nurses will have to transform into intensive care unit nurses due to staffing shortages.^28^ The pandemic caused nurses to experience consistently changing job duties and increased overtime work; yet, nurses exhibited professional solidarity, felt collective empowerment, and activated psychological defense mechanisms.^13^ COVID-19 vaccination campaigns targeted at nurses should be supportive, not punitive, cognizant of ever-present uncertainty and amplified burden that nurses have been balancing during the pandemic.

### Trainees

At the beginning of the pandemic, uncertainty dominated trainees' present and future concerns: potential redeployment to an unplanned department, cancellation of spring rotations, COVID-19 domination of their rotation, burnout, cancellation of annual leave, and concerns about lack of PPE, hot food, and available resting places in the case of long shifts.^29^ Many trainees experienced hopelessness and immense anxiety during the COVID-19 pandemic.^30^ Supervision quality and didactic learning have been disrupted by social distancing measures, whereas credentialing and examinations have been delayed in many disciplines (e.g., radiology).^31^ Many trainees in their fourth year of medical studies were called (by choice or by mandate) immediately to the frontlines during summer 2020 instead of otherwise taking time off.^32^

Releasing HCP trainees to work beyond their established clinical or care competencies is a risk in itself and worsens the ability to objectively evaluate the standardization, adequacy, and evidence-based nature of their training during the COVID-19 pandemic.^32^ In the United States, telemedicine dominates outpatient clinical work, limiting experience with physical examinations, a skill only mastered by practice.^33^ The disproportionate burden placed on the generation of health professional trainees during the COVID-19 pandemic, compared with HCP trained during routine times, poses a significant threat to the future global health workforce.^34^ COVID-19 vaccination campaigns should recognize this and not exclude trainees during vaccine rollout.

### Nonclinical essential workers

Experiences of HCP performing clinical work have dominated headlines regarding HCP experiences on the frontlines of the COVID-19 pandemic. Yet, essential workers within health care settings performing nonclinical duties, often referred to as “support” or “ancillary staff,” have been largely overlooked.^35^ Nonclinical essential workers work in a variety of infrastructure roles within health care, including, but not limited to: maintenance, janitorial, and patient transport services, care assistants, and food transport.^35^ In general, individuals from minority groups, such as Black, Indigenous, and People of Color (BIPOC) populations, are overrepresented across essential nonclinical roles in health care settings.^35^ BIPOC populations have been hit the hardest in the United States by the COVID-19 pandemic, testing positive and dying from COVID-19 at a higher rate than other racial and ethnic groups.^36,37^ Increased burdens of COVID-19 cases and death are documented specifically among working people living in communities with high proportions of people of color and additional social determinants of health, such as poverty, economic segregation, and crowded housing.^38^ Complexity surrounding understanding information regarding COVID-19 prevention, transmission, and infection is oftentimes exacerbated by language barriers, health literacy, conflicting messaging, and mistrust.^35,36,39,40^

Additionally, nonclinical essential workers have had inequitable access to PPE and COVID-19 testing in comparison to their clinical counterparts.^35^ Testing positive for COVID-19 as a nonclinical essential worker from an underrepresented minority group also impacts their individual social needs as well as potentially the social needs of their family, dependents, and community.^35^ Self-isolation may not be possible, and child care may be unaffordable.^35^ Nonclinical essential workers should not continue to be overlooked as HCP COVID-19 vaccine campaigns continue.

## Challenges in COVID-19 Vaccine Uptake Among HCP—Vaccine Hesitancy

Challenges to COVID-19 vaccine uptake among HCP have long been anticipated, particularly in light of established COVID-19 vaccine hesitancy among HCP, a significant contributor to vaccine uptake.^9,41,42^ Vaccine hesitancy exists on a spectrum, ranging from “ultimate refusers” (e.g., anti-vaccination movements) to “ultimate acceptors” (e.g., getting vaccinated without question, doubt), with the majority of people resting somewhere in the middle. Vaccine hesitancy among HCP is particularly unique given HCPs professional duties to care and to “do no harm.”^43^ From a public health perspective, getting vaccinated as an HCP fulfills both professional duties, and refusing vaccination (without a medical or value-based exemption^44^ could contradict, or not sufficiently fulfill, professional duties.

Factors contributing to vaccine hesitancy among HCP include individual or social group influences (i.e., vaccination as a social norm, perceived risk vs. benefit analysis), vaccine and vaccination-specific issues (i.e., vaccine effectiveness, vaccination incentives, access, sufficient training), and contextual influences (i.e., religion, politics, history, socioeconomic status).^45^ Vaccine-refusing HCP are an uncommon but existent minority group. A study investigating vaccine refusal among HCP found that vaccine-refusing HCP have a particularly negatively skewed risk perception against vaccination (i.e., perceived high risk from vaccination or low disease risk), decreased motivation to get vaccinated for the sake of others (i.e., perceived personal and/or financial cost disproportionate to the potential benefit of vaccination), and competing motivational forces (i.e., deciding to act in the interest of protecting oneself vs. caring for others, in light of respective perceived risk perceptions).^46^

COVID-19 vaccine hesitancy among HCP is emerging as vaccination campaigns continue. The fast-tracked nature of COVID-19 vaccines, lacking transparency and information, as well as concerns regarding the political climate in which the vaccine was developed crossed all demographic groups of HCP, regardless of sex, educational level, or ethnicity.^47^ Safety concerns, questions of personal autonomy, and vaccine effectiveness skepticism arise in most cases of vaccine hesitancy.^48,49^ However, the unprecedented speed of development of the vaccine, the emergency pandemic backdrop, significant remaining uncertainty surrounding the vaccine, and the fact that HCP are one of the first groups in the world to get vaccinated (potentially causing many to feel like “guinea pigs”) make HCP hesitancy toward the COVID-19 vaccine nontraditional. Additionally, 40% of HCP in the United States, including both clinical and nonclinical essential workers, are people of color.^50^ Vaccine hesitancy among people of color, particularly in the United States, is an understandable, recognized result of structural systematic racism.^51–53^ Thus far, in the United States, vaccine hesitancy is posing a significant challenge to HCP uptake of the COVID-19 vaccine.^54^

To maximize the benefit of prioritizing HCP to get vaccinated against COVID-19, vaccination campaigns should revisit the complexities of vaccine hesitancy among HCP through engaging in discourse to adjust expectations, provide resources and support, and garner trust accordingly. After all, much vaccine hesitancy coexists with barriers in access to getting vaccinated.

## Challenges in COVID-19 Vaccine Uptake Among HCP—Equity

A clear takeaway from COVID-19 vaccination campaigns across America and globally, regardless of their success: the importance of equity.^55^ Within the United States, access to COVID-19 vaccines and understanding of the process among HCP vary.^56^ At prestigious health care settings, such as academic-affiliated hospitals, nonessential workers such as young researchers and administrative staff not involved in patient care have managed to get vaccinated over many frontline HCP.^57^ Clear disparities emerged between HCP of the same category but in different locations. For example, certain medical students in the United States were able to get vaccinated, whereas others working in different departments or locations were not.^58^

Information regarding COVID-19 vaccine rollout among HCP may be limited to technological means and in the English language, which prevents those who struggle with technology and/or do not have a consistent working smartphone or computer. These struggles may be most prominent among older HCP and nonclinical essential workers.^59^ As a result, they are prevented from staying updated regarding their COVID-19 vaccine access. In settings where confusion and disorder dominates COVID-19 vaccination campaigns, privilege and connections can determine whether or not one will manage to get vaccinated or not, even among prioritized HCP.^57^ After all, in a setting where HCP COVID-19 vaccine access is dependent on registering, as an employee, for a patient portal which they have never used before, HCP may be hesitant to do so. Health care systems and COVID-19 vaccination campaigns need to acknowledge the coexistence of these factors, and ease HCPs access to COVID-19 vaccinations as much as possible.

Although COVID-19 vaccine uptake in the United States among HCP according to occupational groups is currently not widely available, Hall et al. found that among publicly employed HCP in the United Kingdom, doctors had the highest COVID-19 vaccine uptake rate (92%), whereas estates, porters, or security (for the purposes of this article, the equivalent of nonclinical essential workers) had the lowest COVID-19 vaccine uptake rate (82.9%).^11^ Nurses or health care assistants had a COVID-19 vaccine uptake rate of 87%.^11^ Although medical students were not explicitly surveyed in the study, HCP younger than 25 years had a COVID-19 vaccine rate of 83.9%.^11^ Uptake among 80% across all occupational groups is an accomplishment that most health care institutions in the United States would probably celebrate, and although data are currently unavailable, it may be unlikely to achieve, given the gaps in access and decentralization of the American health care system. Regardless, as expected, nonessential clinical workers have the lowest COVID-19 vaccine uptake of the occupational groups. This phenomenon is most likely to be expected across all health care contexts, particularly in the United States, and correlates with equity concerns raised here.

Various systematic interventions and avenues of support could assist in raising COVID-19 vaccine uptake among HCP. Mass vaccination events centrally located in health care settings could create greater awareness and participation in COVID-19 vaccination among HCP, particularly as HCP are under increasing time constraints and pressures as the pandemic continues.^60^ Given the little spare time that HCP have during their shifts, health care systems could provide paid time off, overtime compensation, and childcare to establish clearer access and ability for HCP to get vaccinated against COVID-19.^61^ Nonclinical essential workers in particular are generally nonunionized and therefore have little to no organized “sway” in advocating for receiving such benefits that would assist not only their uptake of the COVID-19 vaccine but also their health and well-being in general.^62^ As a result, we urge health care systems and to take responsibility for proper and equitable HCP access of COVID-19 vaccines within the workplace. This begins with supportive structural interventions, which reduce the burden placed upon individual HCP to understand processes related to COVID-19 vaccine access, from start to finish.

The COVID-19 experiences of HCP at the frontlines of the pandemic are unprecedented, and COVID-19 vaccine rollout is an optimistic step toward the end of the pandemic. Although COVID-19 vaccine hesitancy among HCP exists and is a substantial challenge to COVID-19 vaccine uptake, access determines whether vaccination is a tangible option. Inequities can fuel vaccine hesitancy, particularly among vulnerable neglected populations.^63^ HCP are not immune to inequitable COVID-19 vaccine access. COVID-19 vaccination campaigns should aim to minimize barriers to access, particularly in light of HCPs COVID-19 lived experiences.

## Determining HCP COVID-19 Vaccine Policy

COVID-19 vaccines are not the only vaccines recommended for HCP. The CDC recommends that HCP get vaccinated against hepatitis B, influenza, varicella, and meningococcal disease, as well as the MMR and Tdap vaccines.^64^ Tools used to try and increase vaccination in the past included facilitating access, education, reminders, incentives, signed declination statements, and mandates of different varieties.^65^ Experience with these tools in the contexts of other vaccines suggested that education and other support programs, such as improving access and reminders, increase uptake, but to a limited degree.^65,66^ However, mandates tended to bring compliance to above 95%.^65^ COVID-19 vaccines funded by the U.S. government are, according to officials, free to citizens, so free vaccines may be less relevant, although facilitated access may still be important.^67^

COVID-19 vaccines are different from these other vaccines in several ways. First, the vaccines are authorized under an emergency use authorization (EUA), not through the regular process.^68^ Second, these vaccines use new technology,^69^ and although they went through trials as large or larger than other vaccines, the speed of the trials, combined with the new technology, could cause concern in recipients. Third, the vaccines were authorized in the context of a pandemic, which means both that the stakes were very high, and, as discussed, that HCP were under substantial pressure and stress for a lengthy time. And finally, there is not yet good data that show that COVID-19 vaccines prevent transmission.^70^ Policy options that are justified when there is strong evidence that vaccines prevent transmission may not be justified when it is not.

The legal framework is similar across different HCP groups previously discussed. The starting point is that employers have extensive leeway to set employment conditions, with some caveats discussed below. In the context of vaccines, traditionally, most health care facilities have enforced uniform policies across the workforce, with some differentiating between employees with patient contact and those without. However, we argue that different policies may be suitable for different groups. Three factors should be considered: risk (especially regarding patient contact), financial and status vulnerability, and emotional vulnerability. Higher risk may support stronger policies; but higher vulnerability suggests that supportive policies are more appropriate than coercive ones.

Many health care systems across the world (e.g., in Italy^71^) and institutions within the United States have implemented COVID-19 vaccine mandates for HCP.^72^ We agree with Mello et al.'s proposal that mandates should be an option of last resort, adopted if, and only if, a recommended and accessible vaccine's voluntary uptake has fallen below the level required to prevent spread.^73^ We would further argue that particularly for the more vulnerable populations especially trainees and nonclinical essential workers, a mandate should only be the last resort, with other tools prioritized as much as possible. Instead of implementing hard mandates, where HCP would be terminated from employment without getting vaccinated, employers should consider softer mandates—for example, requiring HCP to wear extra PPE in the case they do not get vaccinated against COVID-19—and incentives to get vaccinated—for example, reduction in health care premiums or time off.

## Legal Consequences of COVID-19 Vaccine Policy

As long as distinctions in policies are not based on protected categories, they are likely legal. Private employers are not subjected to constitutional requirements, but the Civil Rights Act of 1964 forbids discrimination in employment based on race, gender, or religion and may limit those categorizations here. Public employers are subjected to the constitution, including the equal protection clause, but criteria based on categories that are not suspect—for example, based on whether one is a doctor or a resident or a nonclinical employee—are subject to rational basis review. Rational basis review simply requires a reasonable basis for the distinction, linking it to a legitimate government interest—a low bar.^74,75^

That said, any policy with a degree of coercion may be challenged in court, and likely will, and its vulnerability to challenge needs to be considered. Policies focusing on improving access or education, or even requiring that a form be signed upon declining a COVID-19 vaccine, are unlikely to be challenged and have not been challenged in the context of other vaccines. However, mandates have been challenged in the context of other vaccines and may well be challenged here, too.^76,77^ Mandates can be challenged in the context of a unionized workforce if the requirements of collective bargaining were not followed.^76^ As mentioned above, some groups of workers—specifically, nonclinical workers—tend not to be unionized, but other segments—such as nurses and doctors—are often unionized. Mandates can also be challenged under the Americans with Disabilities Act if an employee with a qualifying disability alleges they did not receive the required accommodation.^76^ Mandates can be challenged on the grounds that an employee with sincere religious objections to the vaccine was not provided reasonable accommodation, although the bar for refusing accommodation for religious objections is lower.^77^

Current COVID-19 vaccines, authorized under an EUA only, may face another type of legal challenge. It is unclear whether employers can legally mandate a vaccine under an EUA. The statutory language not only talks about informing individuals that they have the option to refuse or accept a vaccine but also talks about the possibility that individuals will face consequences for such refusal.^78^ The fact that the vaccine is not yet fully approved may make it experimental, and there is an argument that experimental products cannot be required. However, the vaccines are authorized for use by the U.S. Food and Drug Administration (FDA), and traditionally, employers may set work conditions. The EUA statute says nothing about employers or states—it does not explicitly limit the power to require a vaccine. Reading into this vague language, a prohibition for employers to do something employers, traditionally, could legally do (i.e., mandate vaccines), is reading a lot into the text. This is an area of uncertainty, although employers are acting on the assumption that they can mandate these vaccines, and may have good grounds. Before COVID-19 vaccines, the only previous EUA for a vaccine was in 2005, for an anthrax vaccine for the military only—and the military faces a different legal environment than the general population. There are, therefore, no cases on point, although we likely will see cases emerging from the COVID-19 pandemic.

On December 16, 2020, the Equal Employment Opportunity Commission (EEOC) issued guidance related to COVID-19 vaccines.^79^ The guidance made it clear that the EEOC was treating COVID-19 vaccines similarly to other vaccines and mentioned only that the FDA has an obligation to inform recipients about the option to refuse the vaccines and consequences.^79^ Employers mandating COVID-19 vaccines may face legal challenges, but the risk is likely not higher than for other vaccines mandates, and in the health care settings, those often survive scrutiny. Areas employers should be mindful of are the need to negotiate with any unions that represent their workforce, and the need to make available accommodations to those with sincere religious objections or qualifying disabilities, unless accommodating is too high a burden.

## Conclusions

Raising COVID-19 vaccine uptake among HCP has been challenging for a variety of reasons, including confusing distribution campaigns, vaccine hesitancy, and inequity. This analysis evaluated the existing challenges to the implementation of COVID-19 vaccination among different occupational groups of HCP—physicians, nurses, trainees, and nonclinical essential workers—through their lived experiences at the frontlines of the pandemic. To raise COVID-19 vaccine uptake among HCP, health care systems should focus on minimizing inequities inherent in existing campaigns. We suggest that the health care systems and vaccination campaigns provide financial and social support to offset respective burdens.

There are a variety of COVID-19 vaccine policy options targeted to HCP to consider, ranging from recommendations to mandates. COVID-19 vaccine policy making is unique for many reasons, and we argue that mandates should be the last resort for COVID-19 vaccine policy among HCP insofar as all other interventions have been employed. Policymakers should consider involving HCP in the process to provide representation in the decision-making process. After all, HCP have been the backbone of the global response to COVID-19. Vaccinating them against COVID-19 is the ethical duty of health care systems. Health care institutions should recognize the lived experiences of different occupational groups HCP on the frontlines of the pandemic as they further target HCP in COVID-19 vaccination campaigns by improved implementation, which recognizes that not all HCP are alike.

## Abbreviations Used

BIPOC: Black, Indigenous, and People of Color
CDC: Centers for Disease Control and Prevention
COVID-19: coronavirus disease 2019
EEOC: Equal Employment Opportunity Commission
EUA: emergency use authorization
FDA: U.S. Food and Drug Administration
HCP: health care personnel

## References

[1] CDC COVID-19 Response Team. Characteristics of health care personell with COVID-19—United States, February 12–April 9, 2020. 2020;69:477–481. 10.15585/mmwr.mm6915e6 PMC7755055 32298247

[2] Gaynor TS, Wilson ME. Social vulnerability and equity: the disproportionate impact of COVID-19. Public Adm Rev. 2020;80:832–838. 10.1111/puar.13264 PMC7361760 32836465

[3] Lovelace B Jr., Feuer W. Covid vaccine distribution has been slower than U.S. officials thought it would be. CNBC. 2021;Sect. Health & Science. Available at https://www.cnbc.com/2020/12/23/covid-vaccine-distribution-has-been-slower-than-us-officials-thought-it-would-be.html Accessed May 28, 2021.

[4] Dooling K, McClung N, Chamberland M, et al. The Advisory Committee on Immunization Practices' Interim Recommendation for Allocating Initial Supplies of COVID-19 Vaccine—United States, 2020. MMWR Morb Mortal Wkly Rep. 2020;69:1857–1859. 33301429 10.15585/mmwr.mm6949e1 PMC7737687

[5] Beer T. Large numbers of health care and frontline workers are refusing Covid-19 vaccine. Forbes. 2021. Available at https://www.forbes.com/sites/tommybeer/2021/01/02/large-numbers-of-health-care-and-frontline-workers-are-refusing-covid-19-vaccine/?sh=138be113c962 Accessed May 28, 2021.

[6] Inskeep S, Glenn H. At Houston Hospital, Head Of COVID-19 Unit Sees Some Staff Wary Of A Vaccine. NPR. 2020. Available at https://www.npr.org/2020/12/16/947248103/at-houston-hospital-head-of-covid-19-unit-sees-some-staff-wary-of-a-vaccine Accessed May 28, 2021.

[7] Wang J, Zhou M, Liu F. Reasons for healthcare workers becoming infected with novel coronavirus disease 2019 (COVID-19) in China. J Hosp Infect. 2020;105:100–101. 32147406 10.1016/j.jhin.2020.03.002 PMC7134479

[8] Kinman G, Teoh K, Harriss A. Supporting the well-being of healthcare workers during and after COVID-19. Occup Med (Lond). 2020;70:294–296. 32428225 10.1093/occmed/kqaa096 PMC7313855

[9] Dror AA, Eisenbach N, Taiber S, et al. Vaccine hesitancy: the next challenge in the fight against COVID-19. Eur J Epidemiol. 2020;35:775–779. 32785815 10.1007/s10654-020-00671-y PMC8851308

[10] Lucia VC, Kelekar A, Afonso NM. COVID-19 vaccine hesitancy among medical students. J Public Health (Oxf). 2020:fdaa230. DOI: 10.1093/pubmed/fdaa230 PMC7799040 33367857

[11] Hall VJ, Foulkes S, Saei A, et al. COVID-19 vaccine coverage in health-care workers in England and effectiveness of BNT162b2 mRNA vaccine against infection (SIREN): a prospective, multicentre, cohort study. Lancet. 2021;397:1725–1735. 33901423 10.1016/S0140-6736(21)00790-X PMC8064668

[12] Alderwick H, Dixon J. The NHS long term plan. BMJ. 2019;364:l84. 30617185 10.1136/bmj.l84 PMC6350418

[13] Mattila E, Peltokoski J, Neva MH, et al. COVID-19: anxiety among hospital staff and associated factors. Ann Med. 2020;53:237–246. 10.1080/07853890.2020.1862905 PMC7877952 33350869

[14] Chew NWS, Lee GKH, Tan BYQ, et al. A multinational, multicentre study on the psychological outcomes and associated physical symptoms amongst healthcare workers during COVID-19 outbreak. Brain Behav Immun. 2020;88:559–565. 32330593 10.1016/j.bbi.2020.04.049 PMC7172854

[15] Sominsky L, Walker DW, Spencer SJ. One size does not fit all—patterns of vulnerability and resilience in the COVID-19 pandemic and why heterogeneity of disease matters. Brain Behav Immun. 2020;87:1–3. 32205119 10.1016/j.bbi.2020.03.016 PMC7102615

[16] Bansal P, Bingemann TA, Greenhawt M, et al. Clinician wellness during the COVID-19 pandemic: extraordinary times and unusual challenges for the allergist/immunologist. J Allergy Clin Immunol Pract. 2020;8:1781–1790.e3. 32259628 10.1016/j.jaip.2020.04.001 PMC7129776

[17] Ing EB, Xu Q, Salimi A, et al. Physician deaths from corona virus (COVID-19) disease. Occup Med (Lond). 2020;70:370–374. 32409839 10.1093/occmed/kqaa088 PMC7239175

[18] Lemaire JB, Wallace JE. Burnout among doctors. BMJ. 2017;358:j3360. 28710272 10.1136/bmj.j3360

[19] Cabrini L, Grasselli G, Cecconi M. Yesterday heroes, today plague doctors: the dark side of celebration. Intensive Care Med. 2020;46:1790–1791. 32613431 10.1007/s00134-020-06166-4 PMC7328643

[20] Damery S, Draper H, Wilson S, et al. Healthcare workers' perceptions of the duty to work during an influenza pandemic. J Med Ethics. 2009;36:12–18. 10.1136/jme.2009.032821 20026687

[21] Cox CL. ‘Healthcare Heroes': problems with media focus on heroism from healthcare workers during the COVID-19 pandemic. J Med Ethics. 2020;46:510–513. 32546658 10.1136/medethics-2020-106398 PMC7316119

[22] Jackson D, Anders R, Padula WV, et al. Vulnerability of nurse and physicians with COVID-19: monitoring and surveillance needed. J Clin Nurs. 2020;29:3584–3587. 32428345 10.1111/jocn.15347 PMC7276813

[23] Sun N, Wei L, Shi S, et al. A qualitative study on the psychological experience of caregivers of COVID-19 patients. Am J Infect Control. 2020;48:592–598. 32334904 10.1016/j.ajic.2020.03.018 PMC7141468

[24] Prestia AS. The moral obligation of nurse leaders. Nurse Leader. 2020;18:326–328. 32313516 10.1016/j.mnl.2020.04.008 PMC7167551

[25] Schutz V, Shattell M. Impact of COVID-19: what does it mean for nurses and health systems? J Psychosoc Nurs Ment Health Serv. 2020;58:2–3. 10.3928/02793695-20200707-01 32744640

[26] Wagner AL, Masters NB, Domek GJ, et al. Comparisons of vaccine hesitancy across five low- and middle-income countries. Vaccines (Basel). 2019;7:155. 10.3390/vaccines7040155 PMC6963484 31635270

[27] Morley G, Grady C, McCarthy J, et al. Covid-19: ethical challenges for nurses. Hastings Cent Rep. 2020;50:35–39. 10.1002/hast.1110 PMC7272859 32410225

[28] Shanafelt T, Ripp J, Trockel M. Understanding and addressing sources of anxiety among health care professionals during the COVID-19 pandemic. JAMA. 2020;323:2133–2134. 32259193 10.1001/jama.2020.5893

[29] Rimmer A. Covid-19: what do trainees need to know? BMJ. 2020;368:m1276. 32220829 10.1136/bmj.m1276

[30] Shaw SCK. Hopelessness, helplessness and resilience: the importance of safeguarding our trainees' mental wellbeing during the COVID-19 pandemic. Nurse Educ Pract. 2020;44:102780. 32272394 10.1016/j.nepr.2020.102780 PMC7138173

[31] Alvin MD, George E, Deng F, et al. The impact of COVID-19 on radiology trainees. Radiology. 2020;296:246–248. 32216719 10.1148/radiol.2020201222 PMC7233407

[32] Harrington RA, Elkind MSV, Benjamin IJ. Protecting medical trainees on the COVID-19 frontlines saves us all. Circulation. 2020;141:e775–e777. 32250654 10.1161/CIRCULATIONAHA.120.047454 PMC7176273

[33] Edigin E, Eseaton PO, Shaka H, et al. Impact of COVID-19 pandemic on medical postgraduate training in the United States. Med Educ Online. 2020;25:1774318. 32493181 10.1080/10872981.2020.1774318 PMC7448893

[34] Kadhum M, Farrell S, Hussain R, et al. Mental wellbeing and burnout in surgical trainees: implications for the post-COVID-19 era. Br J Surg. 2020;107:e264. 32463518 10.1002/bjs.11726 PMC7283838

[35] Maurer LR, Perez NP, Witt EE, et al. Protecting our own: equity for employees as hospitals battle COVID-19. Health Equity. 2020;4:394–396. 32999949 10.1089/heq.2020.0024 PMC7520652

[36] Thakur N, Lovinsky-Desir S, Bime C, et al. The structural and social determinants of the racial/ethnic disparities in the U.S. COVID-19 pandemic. What's our role? Am J Respir Crit Care Med. 2020;202:943–949. 32677842 10.1164/rccm.202005-1523PP PMC7528789

[37] Berkowitz SA, Wiley Cene C, Chatterjee A. Covid-19 and health equity—time to think big. N Engl J Med. 2020;383:e76. 32706955 10.1056/NEJMp2021209

[38] Krieger N. ENOUGH: COVID-19, Structural racism, police brutality, plutocracy, climate change-and time for health justice, democratic governance, and an equitable, sustainable future. Am J Public Health. 2020;110:1620–1623. 32816556 10.2105/AJPH.2020.305886 PMC7542259

[39] Mantwill S, Monestel-Umana S, Schulz PJ. The relationship between health literacy and health disparities: a systematic review. PLoS One. 2015;10:e0145455. 26698310 10.1371/journal.pone.0145455 PMC4689381

[40] Abuelgasim E, Saw LJ, Shirke M, et al. COVID-19: unique public health issues facing Black, Asian and minority ethnic communities. Curr Probl Cardiol. 2020;45:100621. 32448759 10.1016/j.cpcardiol.2020.100621 PMC7207142

[41] Kwok KO, Li KK, Wei WI, et al. Influenza vaccine uptake, COVID-19 vaccination intention and vaccine hesitancy among nurses: a survey. Int J Nurs Stud. 2020;114:103854. 33326864 10.1016/j.ijnurstu.2020.103854 PMC7831770

[42] Detoc M, Bruel S, Frappe P, et al. Intention to participate in a COVID-19 vaccine clinical trial and to get vaccinated against COVID-19 in France during the pandemic. Vaccine. 2020;38:7002–7006. 32988688 10.1016/j.vaccine.2020.09.041 PMC7498238

[43] van den Hoven M, Verweij M. Professional solidarity: the case of influenza immunization. Am J Bioeth. 2013;13:51–52. 10.1080/15265161.2013.813606 23952837

[44] Blank C, Gemeinhart N, Dunagan WC, et al. Mandatory employee vaccination as a strategy for early and comprehensive health care personnel immunization coverage: experience from 10 influenza seasons. Am J Infect Control. 2020;48:1133–1138. 32238270 10.1016/j.ajic.2020.01.015

[45] Paterson P, Meurice F, Stanberry LR, et al. Vaccine hesitancy and healthcare providers. Vaccine. 2016;34:6700–6706. 27810314 10.1016/j.vaccine.2016.10.042

[46] Betsch C. Overcoming healthcare workers' vaccine refusal—competition between egoism and altruism. Euro Surveill. 2014;19:1–5. 10.2807/1560-7917.es2014.19.48.20979 25496574

[47] Gadoth A, Halbrook M, Martin-Blais R, et al. Assessment of COVID-19 vaccine acceptance among healthcare workers in Los Angeles. medRxiv. 2020. Available at https://www.medrxiv.org/content/10.1101/2020.11.18.20234468v1 Accessed May 28, 2021. 10.7326/M20-7580 PMC7926184 33556267

[48] Karafillakis E, Dinca I, Apfel F, et al. Vaccine hesitancy among healthcare workers in Europe: a qualitative study. Vaccine. 2016;34:5013–5020. 27576074 10.1016/j.vaccine.2016.08.029

[49] Nutman A, Yoeli N. Influenza vaccination motivators among healthcare personnel in a large acute care hospital in Israel. Isr J Health Policy Res. 2016;5:52. 27800154 10.1186/s13584-016-0112-5 PMC5080682

[50] Harmon Courage K. It's essential to understand why some health care workers are putting off vaccination. Vox. 2021. Available at https://www.vox.com/22214210/covid-vaccine-health-care-workers-safety-fears Accessed May 28, 2021.

[51] Egede LE, Walker RJ. Structural racism, social risk factors, and Covid-19—a dangerous convergence for Black Americans. N Engl J Med. 2020;383:e77. 32706952 10.1056/NEJMp2023616 PMC7747672

[52] Warren RC, Forrow L, Hodge DA, Sr., et al. Trustworthiness before trust—Covid-19 vaccine trials and the Black community. N Engl J Med. 2020;383:e121. 33064382 10.1056/NEJMp2030033

[53] Mende-Siedlcki P, Perron S, Gibbons C, et al. Seeing no pain: assessing the generalizability of racial bias in pain perception. Psyarxiv. 2020. Available at https://psycnet.apa.org/record/2021-21105-001 Accessed May 28, 2021. 10.1037/emo0000953 33661666

[54] Robbins R, Tavernise S, Otterman S. Cash, breakfasts and firings: an all-out push to vaccinate wary medical workers. The New York Times. 2021;Sect. COVID-19 Vaccines. Available at https://www.nytimes.com/2021/01/14/business/covid-vaccine-health-hospitals.html Accessed May 28, 2021.

[55] Davies G. Israel is leading the world in its vaccinations, but the program is not without controversy. ABC News. 2021. Available at https://abcnews.go.com/International/israel-leading-world-vaccinations-program-controversy/story?id=75253507 Accessed May 28, 2021.

[56] Price A. A week of vaccine distribution in Texas: sleeping in cars in Mercedes, a jammed call center in Houston and no line at a San Angelo H-E-B. Austin American-Statesman. 2021;Sect. News. Available at https://www.statesman.com/story/news/2021/01/08/texas-covid-19-vaccine-rollout-slow-uneven/6567380002/ Accessed May 28, 2021.

[57] Mandavilli A. At Elite Medical Centers, Even Workers Who Don't Qualify Are Vaccinated. The New York Times. 2021;Sect. Covid-19 vaccines. Available at https://www.nytimes.com/2021/01/10/health/coronavirus-hospitals-vaccinations.html Accessed May 28, 2021.

[58] Goldberg E. Some Medical Students Celebrate With Covid Vaccine Selfies as Others Wait in Line. The New York Times. 2021. Available at https://www.nytimes.com/2021/01/14/health/covid-vaccine-medical-schools.html Accessed May 28, 2021.

[59] Press VG, Huisingh-Scheetz M, Arora V. Op-ed: COVID-19 vaccine registration by app? Confusing technology reveals challenges for seniors using telemedicine. The Chicago Tribune. 2021;Sect. Commentary Opinion. Available at https://www.chicagotribune.com/opinion/commentary/ct-opinion-vaccine-seniors-digital-literacy-telemedicine-20210120-x6mbmd2swjdanouifuqkfmll2q-story.html Accessed May 28, 2021.

[60] Heymann DL, Aylward RB. Mass vaccination: when and why. Curr Top Microbiol Immunol. 2006;304:1–16. 16989261 10.1007/3-540-36583-4_1

[61] Charney RL, Rebmann T, Flood RG. Emergency childcare for hospital workers during disasters. Pediatr Emerg Care. 2015;31:839–843. 26583934 10.1097/PEC.0000000000000629

[62] O'Donnell J. Essential workers during COVID-19: at risk and lacking union representation: Brookings; 2020 [September 3, 2020]. Available at https://www.brookings.edu/blog/up-front/2020/09/03/essential-workers-during-covid-19-at-risk-and-lacking-union-representation/ Accessed May 28, 2021.

[63] Leask J, Willaby HW, Kaufman J. The big picture in addressing vaccine hesitancy. Hum Vaccin Immunother. 2014;10:2600–2602. 25483479 10.4161/hv.29725 PMC4975059

[64] CDC. Recommended Vaccines for Healthcare Workers: Centers for Disease Control and Prevention; 2020. Available at https://www.cdc.gov/vaccines/adults/rec-vac/hcw.html Accessed May 28, 2021.

[65] Hollmeyer H, Hayden F, Mounts A, et al. Review: interventions to increase influenza vaccination among healthcare workers in hospitals. Influenza Other Respir Viruses. 2013;7:604–621. 22984794 10.1111/irv.12002 PMC5781006

[66] Stead M, Critchlow N, Patel R, et al. Improving uptake of seasonal influenza vaccination by healthcare workers: implementation differences between higher and lower uptake NHS trusts in England. Infect Dis Health. 2019;24:3–12. 30541694 10.1016/j.idh.2018.09.082

[67] CDC. Frequently Asked Questions about Covid-19 Vaccinaion: Centers for Disease Control and Prevention; 2021 [updated February 6, 2021]. Available at https://www.cdc.gov/coronavirus/2019-ncov/vaccines/faq.html Accessed May 28, 2021.

[68] Bauchner H, Malani PN, Sharfstein J. Reassuring the public and clinical community about the scientific review and approval of a COVID-19 vaccine. JAMA. 2020;324:1296–1297. 32910154 10.1001/jama.2020.18860

[69] Jackson LA, Anderson EJ, Rouphael NG, et al. An mRNA vaccine against SARS-CoV-2—preliminary report. N Engl J Med. 2020;383:1920–1931. 32663912 10.1056/NEJMoa2022483 PMC7377258

[70] Kim JH, Marks F, Clemens JD. Looking beyond COVID-19 vaccine phase 3 trials. Nat Med. 2021;27:205–211. 33469205 10.1038/s41591-021-01230-y

[71] Paterlini M. Covid-19: Italy makes vaccination mandatory for healthcare workers. BMJ. 2021;373:n905. 33824155 10.1136/bmj.n905

[72] Gooch K. Houston Methodist implements mandatory COVID-19 vaccinations. Becker's Hospital Review. 2021. Available at https://www.beckershospitalreview.com/workforce/houston-methodist-implements-mandatory-covid-19-vaccinations.html Accessed May 28, 2021.

[73] Mello MM, Silverman RD, Omer SB. Ensuring uptake of vaccines against SARS-CoV-2. N Engl J Med. 2020;383:1296–1299. 32589371 10.1056/NEJMp2020926

[74] City of Cleburne v. Cleburne Living Ctr.: 473 U.S.; 1985. p. 432, 42. Available at https://supreme.justia.com/cases/federal/us/473/432/ Accessed May 28, 2021.

[75] San Antonio Indep. Sch. Dist. v. Rodriguez. 411 US; 1973. p. 1, 35. Available at https://supreme.justia.com/cases/federal/us/411/1/ Accessed May 28, 2021.

[76] Najera RF, Reiss DR. First do no harm: protecting patients through immunizing health care workers. Health Matrix. 2016;26:363–402. 27263256

[77] Opel DJ, Sonne JA, Mello MM. Vaccination without litigation—addressing religious objections to hospital influenza-vaccination mandates. N Engl J Med. 2018;378:785–788. 29384718 10.1056/NEJMp1716147

[78] 21 U.S.C. 564 (e)(1)(A)(ii)(iii). Available at https://scholarlycommons.law.case.edu/healthmatrix/vol26/iss1/13/ Accessed May 28, 2021.

[79] Commission USEEO. What You Should Know About COVID-19 and the ADA, the Rehabilitation Act, and Other EEO Laws 2020 [updated December 16, 2020]. Available at https://www.eeoc.gov/wysk/what-you-should-know-about-covid-19-and-ada-rehabilitation-act-and-other-eeo-laws. Accessed May 28, 2021.

